# Discovery of a Novel Seminal Fluid Microbiome and Influence of Estrogen Receptor Alpha Genetic Status

**DOI:** 10.1038/srep23027

**Published:** 2016-03-14

**Authors:** Angela B. Javurek, William G. Spollen, Amber M. Mann Ali, Sarah A. Johnson, Dennis B. Lubahn, Nathan J. Bivens, Karen H. Bromert, Mark R. Ellersieck, Scott A. Givan, Cheryl S. Rosenfeld

**Affiliations:** 1 Bond Life Sciences Center, University of Missouri, Columbia, MO 65211 USA; 2 Biomedical Sciences, University of Missouri, Columbia, MO 65211 USA; 3 Informatics Research Core Facility, University of Missouri, Columbia, MO 65211 USA; 4 Biochemistry, University of Missouri, Columbia, MO 65211 USA; 5 MU Center for Botanical Interaction Studies, University of Missouri, Columbia, MO 65211 USA; 6 Animal Sciences, University of Missouri, Columbia, MO 65211 USA; 7 Child Health, University of Missouri, Columbia, MO 65211 USA; 8 Genetics Area Program, University of Missouri, Columbia, MO 65211 USA; 9 DNA Core Facility, University of Missouri, Columbia, MO 65211 USA; 10 Agriculture Experimental Station-Statistics, University of Missouri, Columbia, MO 65211 USA; 11 Molecular Microbiology and Immunology, University of Missouri, Columbia, MO 65211 USA; 12 Thompson Center for Autism and Neurobehavioral Disorders, University of Missouri, Columbia, MO 65211 USA

## Abstract

Bacteria harbored in the male reproductive system may influence reproductive function and health of the male and result in developmental origins of adult health and disease (DOHaD) effects in his offspring. Such effects could be due to the seminal fluid, which is slightly basic and enriched with carbohydrates; thereby, creating an ideal habitat for microbes or a potential seminal fluid microbiome (SFM). Using wild-type (WT) and estrogen receptor-alpha (ESR1) knockout (KO) male mice, we describe a unique SFM whose inhabitants differ from gut microbes. The bacterial composition of the SFM is influenced according to whether mice have functional *Esr1* genes. *Propionibacterium acnes*, causative agent of chronic prostatitis possibly culminating in prostate cancer, is reduced in SFM of ESR1 KO compared to WT mice (P ≤ 0.0007). In certain genetic backgrounds, WT mice show a greater incidence of prostate cancer than ESR1 KO, which may be due to increased abundance of *P. acnes*. Additionally, select gut microbiome residents in ESR1 KO males, such as Lachnospiraceae and Christensenellaceae, might contribute to previously identified phenotypes, especially obesity, in these mutant mice. Understanding how genetics and environmental factors influence the SFM may provide the next frontier in male reproductive disorders and possibly paternal-based DOHaD diseases.

The future health of offspring can be influenced by the condition of the male parent^1^,^2^,^3^,^4^,^5^,^6^,^7^,^8^,^9^. For example, paternal obesity in mice has been linked to delayed development, altered carbohydrate utilization, and mitochondrial disturbances^4^,^5^ ando various other health effects, such as obesity, cardiovascular and reproductive disorders in F_1_ pups^1^,^2^,^6^,^7^,^8^,^9^,^10^,^11^,^12^,^13^,^14^,^15^. Analogous effects have been noted in humans^16^,^17^,^18^, but in neither species has the mechanism of transmission to the offspring been defined.

Paternal condition has been proposed to give rise to F_1_ offspring developmental origins of health and disease (DOHaD) and trans-generational (transmission to future descendants) effects through three mechanisms. The first is direct effects on the epigenome of male germ cells, such as alterations in DNA methylation status^19^ and microRNAs (miRs), piRNAs, enhancer-derived RNAs (eRNAs), and tRNA fragments (tRFs)^20^,^21^,^22^,^23^. The latter may be produced and released by epithelial cells of the epididymis and then taken-up by the spermatozoa as they mature. Histone protein modifications in sperm may also lead to phenotypic alterations in F_1_ offspring and F_2_ descendants^24^.

A second proposed mechanism for paternal transmission of information to the F_1_ generation is via seminal fluid content, which could impact female reproductive tract physiology and conceptus development^3^. A third proposed possible mechanism is that the female responds to the perceived fitness of her partner by modulating her investment in his offspring^25^,^26^,^27^. In a recent review, it has been speculated that fathers may transmit information via microbiota to their partners and progeny^20^. The most likely source of such microbiota is the seminal vesicles.

Almost all mammalian males, except carnivores, possess seminal vesicles (also termed vesicular glands). These glands are simple tubular and situated below the urinary bladder and vas deferens. In humans, approximately 50–70% of the seminal fluid is produced by the seminal vesicles^28^. This fluid is slightly basic (pH < 7.2) and contains fructose, proteins, enzymes, mucus, vitamin C, flavins, phosphorylcholine, and prostaglandins. Fructose is thought to provide the primary source of metabolic energy for the spermatozoa, but this nutrient can also be utilized by microorganisms, such as fructophilic lactic acid bacteria (FLAB). Therefore, the seminal vesicles could provide a unique niche for such microorganisms to thrive^29^. The seminal vesicles also express ESR1 and 2, suggesting that their secretory activity might be affected by endogenous and environmental estrogenic hormones^30^,^31^,^32^,^33^,^34^,^35^. Experiments performed on mice null for *ESR1* and *ESR2* also implicate estrogens in regulating the function of the seminal glands^36^,^37^,^38^.

Bacteria are present in semen samples of men and can be transmitted to their female sexual partners^39^,^40^,^41^,^42^,^43^,^44^,^45^,^46^, but it is unclear whether these bacteria originate from colonies established in the seminal vesicles or elsewhere in the male reproductive and urinary tract systems. To address whether the seminal vesicles harbor a distinct microbiome and whether the composition of this population of microorganisms can be potentially influenced by estrogens, we collected the seminal fluid and tissue from ESR1 KO and wild-type (WT) male mice. Fecal samples from the same animals were collected to determine the extent to which the putative SFM resembled that of the large bowel.

## Results

### Comparison of the seminal fluid microbiome to the fecal microbiome

**Figure 1:**
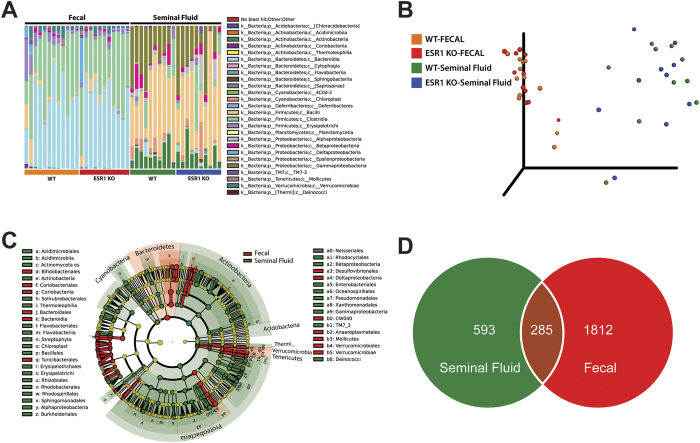
Comparison of the fecal microbiome to the seminal fluid microbiome. (**A**) Bar plot of the most abundant bacterial classes between fecal and seminal fluid samples of WT and ESR1 KO mice. Replicates: WT fecal = 12; ESR1 KO fecal = 11; WT Seminal Fluid = 10; ESR1 KO Seminal Fluid = 10. While we collected fecal and seminal fluid samples from all animals, for reasons that are not clear one of the fecal and one of the seminal fluid samples did not sequence properly. Thus, these results were not considered in the analysis, and this is the reason for the disparate number of samples. (**B**) PCoA of fecal and seminal fluid samples from WT and ESR1 KO mice. The fecal samples from WT (orange circles) and ESR1 KO (red circles) clustered separately from the seminal fluid samples of WT (green circles) and ESR1 KO (blue circles) males (p = 0.0001 by PERMANOVA). (**C**) Cladogram derived from LEfSe analysis of 16S sequences from WT and ESR1 KO fecal and seminal fluid samples. Green shaded areas indicate bacterial orders that more consistently describe the seminal fluid environment; whereas, red shaded areas indicate those that more consistently describe the fecal environment. This diagram provides an estimate of the OTUs that characterize the fecal and seminal fluid microbiome samples. (**D**) Venn diagram comparison of OTUs that overlap between seminal fluid and fecal samples and those only present in seminal fluid or fecal samples.

The first goal was to ascertain whether the seminal fluid harbored a unique microbiome. By sequencing the V4 region of the 16S rRNA gene on the Illumina MiSeq, several microbiota were identified in the seminal fluid of WT and ESR1 KO males. Collectively, they comprise a seminal fluid microbiome (SFM, Fig. 1). After identifying the presence of a SFM, we sought to determine whether the microbial community in this region differed from that present in the fecal samples of the same genotype mice. We chose this comparison as the gut or fecal microbiome is one of the best characterized to date.

When the 16S rRNA sequencing results were compared by using Greengenes Version 13_8 (which is available through QIIME, http://qiime.org/home_static/dataFiles.html ftp://greengenes.microbio.me/greengenes_release/gg_13_5/gg_13_8_otus.tar.gz) clear distinctions were evident in the various bacterial classes when comparing fecal to seminal fluid samples (Fig. 1A). A PCoA analysis and cladogram further confirmed that the classes of bacteria inhabiting these two biological samples clustered separately (Fig. 1B and C, p = 0.0001 by PERMANOVA). The Venn diagram revealed that of 2690 operational taxonomic units (OTUs) only 285 were common to both compartments. The seminal fluid contained 593 OTUs not found in the fecal samples (Fig. 1D).

Alpha-diversity was analyzed by plotting and comparing the Chao1 and Shannon-Weiner indices for each sample type (Supplementary Fig. 1). Additionally, rarefaction metrics were plotted and compared between the two sample types. Taken together, these data indicate that the seminal fluid microbial community is somewhat less species-rich and less diverse than the fecal community.

**Figure 2:**
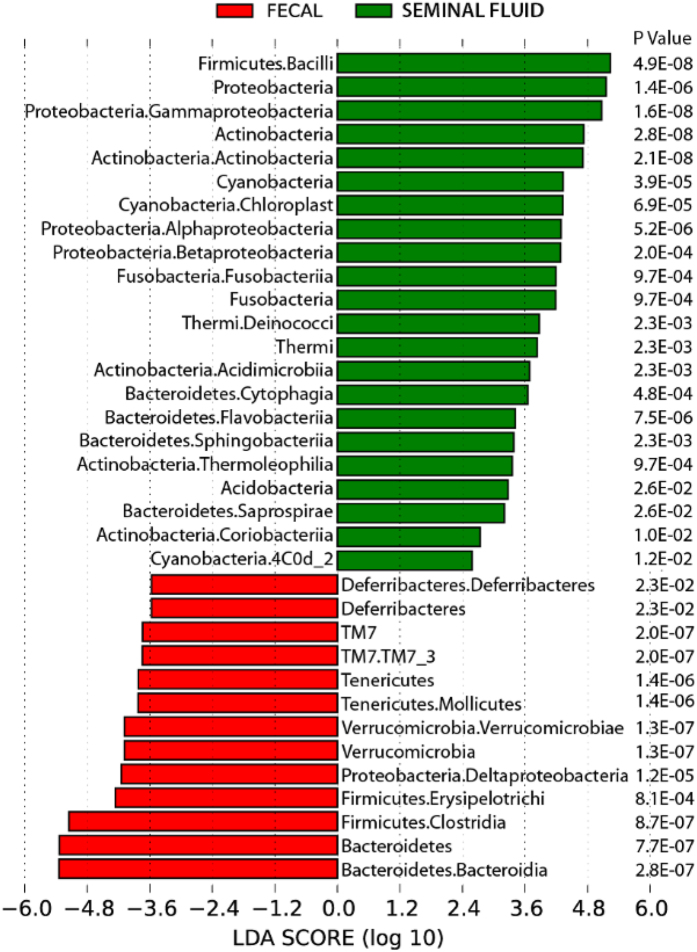
LEfSe comparison of microbiota in seminal fluid or fecal samples. The genera listed in green most consistently describe seminal fluid. In contrast, those listed in red most consistently describe fecal samples. P values are listed by genera.

LEfSe comparison (Fig. 2) of microbiota in the seminal fluid or fecal samples also emphasizes the uniqueness of the seminal fluid microbiome. The genera listed in green, most consistently describe seminal fluid. In contrast, those listed in red are the signature of fecal samples. The SFM was characterized by a preponderance of Bacilli, Proteobacteria, Actinobacteria, Fusobacteria, Flavobacteria, and Acidobacteria (P ≤ 0.05, Fig. 2). In contrast, Defferibacteres, Enericutes, Verrucomicrobia, Deltaproteobacteria, Eryspelotrichi, Closteridia, and Bacteroidetes are more consistently present in the fecal samples compared to the seminal fluid samples (P ≤ 0.05, Fig. 2). Taken together, the data strongly support the finding that the seminal fluid harbors a unique microbiome relative to that present in the fecal samples.

**Figure 3:**
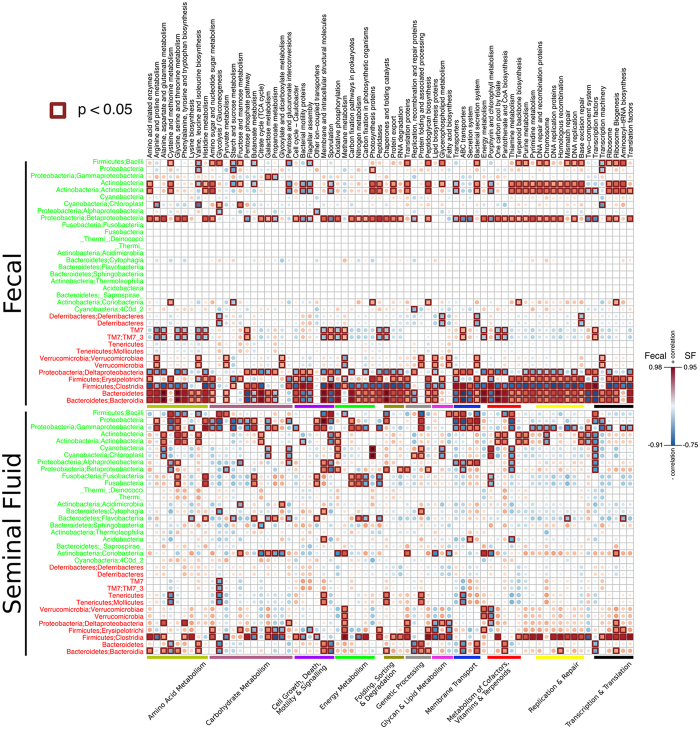
Bacterial metabolic and other pathway differences in the fecal microbiome *vs.* the SFM. As described in Fig. 7 of Ma *et al.*^66^, correlations between the PICRUSt-generated functional profile and QIIME-generated genus level bacterial abundance were calculated and plotted against genotype status for the seminal fluid samples in WT and ESR1 KO mice. Those genera that were identified by LEfSe as being different between the two biological samples are depicted. Metabolic pathway designations are delineated at the bottom of the figure. Shading intensity and size of the circles indicates the Kendall rank correlation coefficient between matrices. Orange/red indicates a positive correlation; whereas blue designates a negative correlation. Red squares surrounding the circles are indicative of a P value ≤ 0.05. As in Fig. 2, genera listed in green were indicative of seminal fluid. In contrast, those listed in red were indicative of fecal samples.

KEGG metabolic pathways correlating with fecal and seminal fluid microbiome differences are listed in Supplementary Data 1, and select examples are illustrated in Fig. 3. Based on the classes that differed between the fecal and SFM and KEGG correlation analyses, it is likely that the disparities in microbial composition noted above, would also contribute to a distinct metabolic milieu in seminal fluid, including changes in amino acids, carbohydrates, energy, glycan and lipid metabolites, cofactors, vitamins, and terpenoids metabolites, that could in turn impact host cell functions.

### Influence of age on the seminal fluid and fecal microbiomes

As part of these studies, we screened various ages of adult WT and ESR1 KO mice (Supplementary Fig. 2A, where the fecal and seminal fluid microbiomes are organized by age of the animals). This was done to increase the likelihood of detecting a SFM, should it exist. To determine if age altered the SFM and fecal microbiome, PCoA and PERMANOVA analysis were done based on age for both microbiomes. Age did not alter the SFM composition (Supplementary Fig. 2B, p = 0.4 by PERMANOVA). However, the fecal microbiome was altered by age (Supplementary Fig. 2C, p = 0.01 by PERMANOVA). To determine which OTUs differed by age, metagenomeSeq^47^ was performed. Thirty OTUs differed based on age. Top genera that were increased in older compared to young males include *Lactobacillus*, Peptostreptococcaceae, *Bifidobacterium pseudolongum*, Clostridiaceae, and Mollicutes (Supplementary Table 1). In contrast, Lachnospiraceae, Dehalobacterium, *Bacteroides acidifaciens*, *Oscillospira*, and another Peptococcaceae were more abundant in younger than older animals.

### Influence of estrogen receptor 1 gene status on the seminal fluid microbiome

**Figure 4:**
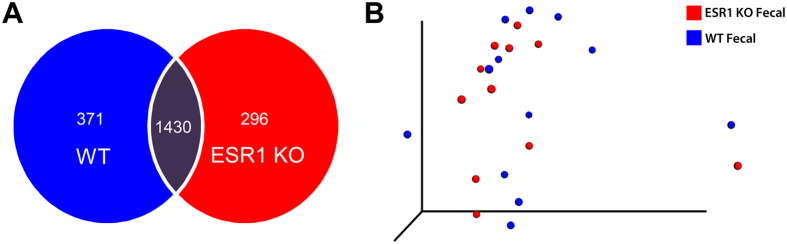
Comparison of the seminal fluid microbiome (SFM) in WT and ES1 KO males. (**A**) Venn diagram comparison of the OTUs that overlap in WT and ESR1 KO mice and those that were unique to each genotype. (**B**) PCoA of the seminal fluid microbiome in WT (blue circles) and ESR1 KO (red circles) mice (p = 0.62 by PERMANOVA). While the Venn diagram comparison reveals that there are differences in the SFM between WT and ESR1 KO animals, they did not result in distinct clustering between these two genotypes. Consequently, metagenomeSeq^47^ was used to determine which OTUs differed between WT and ESR1 KO males (Table 1).

We next investigated whether ESR1 gene status had an impact on the microbiota present in the seminal fluid samples. While the majority of OTUs occurred in both the seminal fluid of WT and ESR1 KO males, each genotype also possessed genera not present in the other genotype (Fig. 4A), although these differences were insufficient to lead to statistically significant clustering of the samples, as illustrated in the PCoA analysis (Fig. 4B). To investigate the potentially subtle microbiome differences in the seminal fluid of these two genotypes, we used metagenomeSeq^47^. This analysis identified several genera that characterized the SFM of WT or ESR1 KO males (Table 1). For example, *Propionibacterium acnes*, was much more prevalent in the seminal fluid of WT then ESR1-/- mice, p = 0.0007. Other genera more plentiful in the seminal fluid of WT males included *Acinetobacter*, *Corynebacterium*, *Streptophyt*a, *Staphylococcus*, and Neisseriaceae. In ESR1 KO mice, *Turicibacter*, *Rhodocyclaceae*, *Streptococcus*, and *Xanthomonadaceae* were much more abundant than in the seminal fluid of WT mice. These differences would also be expected to influence the metabolic environment of the fluid.

**Figure 5:**
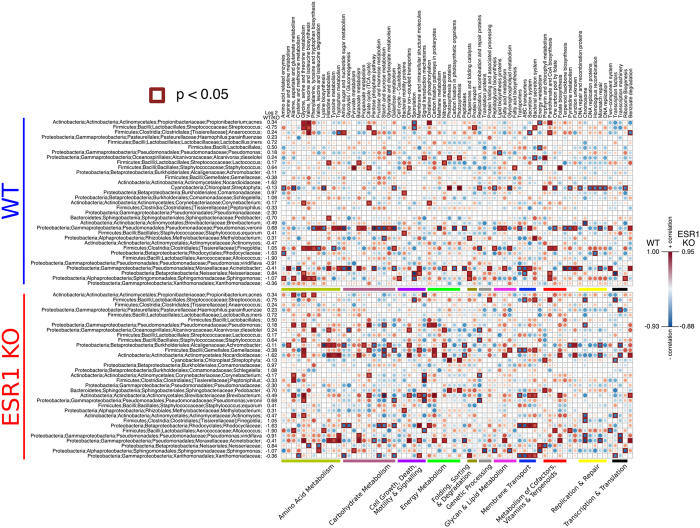
Bacterial metabolic and other pathway differences in the seminal fluid samples of WT *vs.* ESR1 KO mice. As described in Fig. 7 of Ma *et al.*^66^, correlations between the PICRUSt-generated functional profile and QIIME-generated genus level bacterial abundance were calculated and plotted against genotype status for the seminal fluid samples in WT and ESR1 KO mice. Those genera that metagenomeSeq identified as being different between the two genotypes are depicted. Metabolic pathway designations are delineated at the bottom of the figure. Shading intensity and size of the circles indicates the Kendall rank correlation coefficient between matrices. Orange/red indicates a positive correlation; whereas blue designates a negative correlation. Red squares surrounding the circles are indicative of a P value ≤ 0.05. To aide in interpretation of the figure, the log2 values listed in Table 1 are included alongside the genera. Negative values indicate that the OTU is greater in the SFM of ESR1 KO males; whereas, positive values are greater in SFM of WT animals. The same number of replicates was analyzed for these data as listed in Fig. 1.

Based on the bacteria that differed, correlation analyses were performed for various KEGG metabolic and other pathways (Fig. 5 and Supplementary Data 2). In WT males, increased abundance of *P. acnes* was positively correlated with an increase in the abundance in oxidative phosphorylation, several amino acids, pantohenate and CoA biosynthesis. In contrast, sporulation and energy metabolism were negatively correlated with increased abundance of *P. acnes*. Other genera resulting in significant positive and negative metabolic pathway correlations in the seminal fluid of WT males included *Streptococcus*, *Anaerococcus*, *Lactococcus*, *Staphylococcus*, *Pseudomonas veronii*, *Finegoldia*, *Acinetobacter*, Neisseriaceae, and *Sphingomonas*.

In ESR1 KO males, several metabolic pathway alterations correlated with microbiome changes in the seminal fluid. In this group, increased abundance of *P. acnes* positively correlated with several amino acid metabolic pathways but negatively correlated with signal transduction mechanisms. Other genera resulting in significant positive and negative metabolic pathway correlations in the seminal fluid of ESR1 KO males included Streptococcus, *Haemophilus parainfluenzae*, *Pseudomonas*, *Alcanivorax diselolei*, *Staphylococcus*, *Achromobacter*, Gemellaceae, Nocardioidaceae, *Corynebacterium*, *Peptoniphilus*, *Brevibacterium*, *Pseudomonas veronii*, *Actinomyces*, *Alloiococcus*, *Pseudomonas viridiflava*, *Acinetobacter*, Neisseriaceae, *Sphingomonas*, and Xanthomonadaceae.

### Influence of estrogen receptor 1 gene status on the fecal microbiome

**Figure 6:**
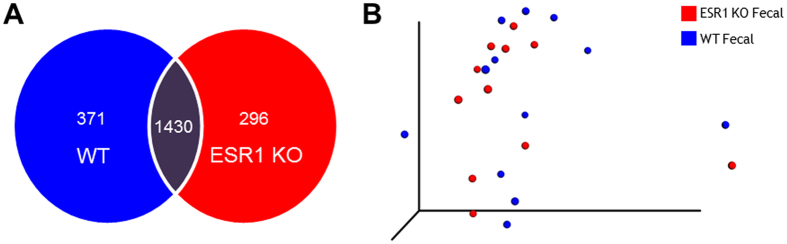
Comparison of the fecal microbiome (SFM) in WT and ES1 KO males. (**A**) Venn diagram comparison of the OTUs that overlap in WT and ESR1 KO mice and those that unique to each genotype. (**B**) PCoA of the fecal microbiome in WT (blue circles) and ESR1 KO (red circles) mice (p = 0.83 by PERMANOVA). While the Venn diagram comparison reveals that there are differences in the SFM between WT and ESR1 KO animals, they did not result in distinct clustering between these two genotypes. Consequently, metagenomeSeq^47^ was used to determine which OTUs differed between WT and ESR1 KO males (Table 2).

As with the seminal fluid results, the fecal samples from WT and ESR1 KO males had more OTUs in common than were unique to each genotype (Fig. 6A). However, these differences did not lead to distinct clusters for each genotype in cluster analysis, as illustrated in Fig. 6B. Analysis with metagenomeSeq^47^ revealed several OTUs that differed in the fecal samples of WT compared to ESR1 KO males. The fecal samples of WT males were enriched with *Ruminococcus*, *Dehalobacterium*, *Dorea*, *Sutterella*, and *Oscillospira*; whereas, *Parabacteroides, Coprococcus*, and *Clostridium* were greater in the fecal samples of ESR1 KO males (Table 2).

**Figure 7:**
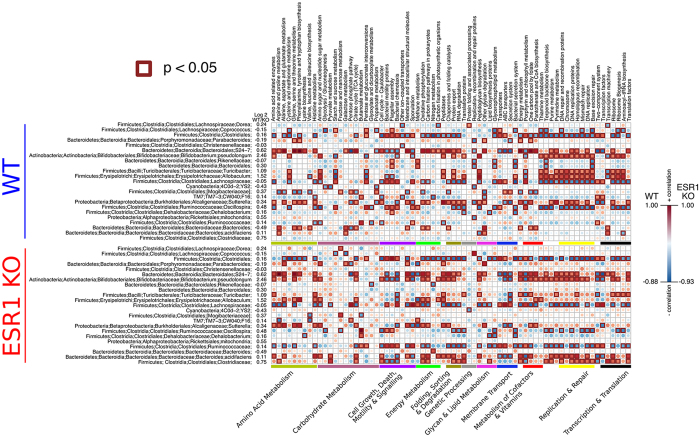
Bacterial metabolic and other pathway differences in the fecal samples of WT *vs.* ESR 1 KO mice. As described in Fig. 7 of Ma *et al.*^66^, correlations between the PICRUSt-generated functional profile and QIIME-generated genus level bacterial abundance were calculated and plotted against genotype status for the fecal samples in WT and ESR1 KO mice. Those genera that metagenomeSeq identified as being different between the two genotypes are depicted. Metabolic pathway designations are delineated at the bottom of the figure. Shading intensity and size of the circles indicates the Kendall rank correlation coefficient between matrices. Orange/red indicates a positive correlation; whereas blue designates a negative correlation. Red squares surrounding the circles are indicative of a P value ≤ 0.05. Negative values indicate that the genera are greater in the fecal microbiome of ESR1 KO males; whereas, positive value are greater in the fecal microbiome of WT animals. The same number of replicates was analyzed for these data as listed in Fig. 1.

The fecal microbiome changes in WT and ESR1 KO were correlated with alterations in several metabolic and other pathways (Fig. 7 and Supplementary Data 3). In WT, the genera that resulted in the most pronounced correlations included *Coprococcus*, *Clostridiales*, Bacteroidales, *Bifidobacterium pseudolongum*, *Turicibacter*, *Allobaculum*, Lachnospiraceae, Mogibacteriaceae, *Sutterella*, *Oscillospira*, Ruminococcaceae, *Bacteroides acidifaciens* and sp. Those that resulted in the greatest associations in ESR1 KO males were Clostridiales, *Parabacteroides*, Bacteroidales, *Bifidobacterium pseudolongum*, *Turicibacter*, *Allobaculum*, Lachnospiraceae, *Sutterella*, *Oscillospira*, *Dehalobacterium*, Ruminococcaceae, *Bacteroides acidifaciens*, and Clostriaceae.

## Discussion

Speculation exists as to whether the seminal vesicles harbor a unique microbiome^20^. The current studies confirm that they do so, at least in mice. The occupants of the SFM are distinct from those residing in the fecal microbiome of these mice. This finding is not surprising as the microhabitat, including nutrient substrate availability, within the each of these biological samples differs immensely. Therefore, the microbiomes present within each of these environments have seemingly evolved over time to the specific qualities of each niche. The bacterial phyla that dominate in the seminal fluid, including Proteobacteria, Actinobacteria, Fusobacteria, and Firmicutes may be able to utilize fructose and other carbohydrates produced by the seminal fluid glands as energy sources. Quantitative PCR or gram staining may be considered as follow-up procedures to confirm some of the OTUs identified in the SFM. Future work should also consider whether other organs of the genitourinary system, including the other accessory sex glands, are colonized by other types of bacteria.

Prior studies have identified bacteria in semen samples from men and were presumed to come from the seminal vesicles^39^,^40^,^41^,^42^,^43^,^44^,^45^,^46^. However, they could have originated from anywhere in the urogenital tract. Examples of bacteria identified in the semen in these other reports include *Peptoniphilis*, *Anaerococcus*, *Finegoldia*, *Peptostreptococcus* spp, *Staphylococcus*, *Streptococcus*, *Corynebacterium*, *Enterococcus*, *Lactobacillus*, *Gardenella*, *Prevotella*, and *Escherichia coli*.

Comparison of genera upregulated in the seminal fluid of ESR1 KO or WT males revealed genotypic differences. There were several genera in common with prior studies^39^,^40^,^41^,^42^,^43^,^44^,^45^,^46^ with semen from men including *Peptoniphilus* (greater in ESR1 KO), *Anaerococcus* (greater in WT), *Finegoldia* (greater in WT), *Staphylococcus* (greater in WT), *Streptococcus* (greater in ESR1 KO), *Corynebacterium* (greater in ESR1 KO), and *Lactobacillus* (greater in WT). Based on our current findings, it suggests that genetic background, at least in mice, can influence bacterial colonies in the SFM. One genera discovered in the SFM that was not identified in these earlier reports is *P. acnes*, which is the causative agent in men and inoculated rodents for chronic prostatitis that can culminate in prostate cancer^48^,^49^. Considering these data, seminal fluid may serve as a storage pool for *P. acnes*, whereupon it may incite infection or cancer only after reaching the prostate gland. As Table 1 illustrates, *P. acnes* is more plentiful in WT compared to ESR1 KO males. WT and ESR1 KO mice typically do not develop prostate cancer. However, when they are bred with TRansgenic Adenocarcinoma of Mouse Prostate (TRAMP) mice, differences emerge in prostate cancer incidence^50^. ESR1 KO/TRAMP mice were less likely to develop aggressive prostate cancer compared to WT/TRAMP mice (5% vs. 19% incidence rate). Notably, current findings suggest that the decreased incidence of prostate cancer in ESR1 KO mice^50^ might be due to the lower amounts of *P. acnes* present in their seminal fluid. The Slusarz *et al.*^50^ study also revealed that ESR2 KO/TRAMP mice had the highest incidence (41%) of prostate cancer. The ESR2 gene was originally identified in the prostate gland^51^. We are in the process of characterizing the SFM in ESR2 KO mice. The prediction is that this group will even have greater abundance of *P. acnes* compared to control mice.

The microbiome alterations in the seminal fluid may also induce changes in virulence factors and the metabolic milieu in these secretions. As shown in Fig. 5, *P. acnes*, *Streptophyta* spp, *Corynebacterium* spp, *Pseudomonas veronii*, and *Acinetobacter* spp in WT males and *P. acnes*, *H. parainfluenzae*, *Acinetobacter* spp, and Sphingomonas are positively associated with significant amounts of metabolic pathway changes in the seminal fluid. Thus, some of the previously identified diet-induced metabolite changes in the seminal fluid may represent a combination of bacterial and host metabolites^3^. The former may influence male reproductive function and overall health (including risk for prostate cancer in the case of *P. acnes*). The genome of *P. acnes* encodes all of the essential components of oxidative phosphorylation^52^. In WT males, increased abundance of this bacterium is associated with greater oxidative phosphorylation metabolic pathways, which may increase the amount of reactive oxygen species, damaging free radicals, and increase the risk for diseases, including prostate cancer. Additionally, transfer of the microbiome to a reproductive partner could influence her reproduction/health, as well as that of the resulting offspring, and even more distant descendants. Therefore, a better understanding of the SFM and how it might be influenced by intrinsic and extrinsic factors is essential.

The microbiomes in fecal samples from WT and ESR1 KO were analyzed in these studies primarily to determine how they compared to the SFM. However, our findings may also partially explain some of the prior phenotypic studies with these animals. For instance, ESR1 KO mice demonstrate obesity, adipocyte hyperplasia and hypertrophy, insulin resistance, and impaired glucose tolerance^53^. At the time, these and other phenotypic disruptions identified in this transgenic mouse model were presumed to be mediated by direct ablation of the ESR1 gene. However, gut dysbiosis can result in metabolic, neurological, and other disease states^54^,^55^,^56^,^57^,^58^. For instance, two Lachnospiraceae were increased in the fecal samples of ESR1 KO males (Table 2), and gut colonization by this bacterial family is associated with increased body weight and hyperglycemia in germ-free *ob/ob* mice^59^. Christensenellaceae is another bacterium augmented in the stool of this group. Increased abundance of this bacterial family positively correlates with body weight and food intake^60^. Administration of an obesogenic diet to mice is associated with an increase in Rikenellaceae^61^,which was significantly greater in ESR1 KO males. Thus, ESR1 KO mice demonstrate genera in their fecal microbiome previously associated with obesity. As with other studies in this area, cause and effect are not certain. For instance, do the gut microbial changes and their products, virulence factors and metabolites underpin the metabolic disruptions, as has been suggested^54^,^55^,^58^ ? Alternatively, do the metabolic disturbances change the nutrient availability and thereby induce shifts in the gut microbiota?

While the SFM did not change throughout adulthood in WT or ESR1 KO animals, the fecal microbiome showed differences with increasing age. Other studies with even older rodent models have shown that age can affect the gut microbiome^62^,^63^. Langille *et al.*^63^ reported that the top overexpressed OTU in the gut microbiome of older mice was Rikenellaceae. In the current studies, this bacterial family was also significantly increased in older compared to young adult animals (Supplementary Table 1). Bacterial shifts in the gut microbiome may occur with age due to alterations in host metabolites, hormones, gastrointestinal structure and physiology, or other intrinsic properties. Conversely, the environment and nutrient composition within the seminal fluid may remain more stable with age, and thus, less prone to bacterial fluctuations.

In conclusion, we have made the novel discovery that the seminal vesicles harbor a unique microbiome. The microbial composition of the SFM can be affected by whether mice possess functional *Esr1* genes. Microbiome shifts in the SFM and the potential changes in the abundance of concomitant metabolic pathways may impact male reproduction and health, his female reproductive partners, offspring, and future descendants. Thus, understanding the SFM and how the resident populations may be influenced by genetic background and environmental factors may spawn future research in biomedical sciences. Increased abundance of *P. acnes* and associated metabolic pathways in the seminal fluid of WT animals might render them at greater risk for prostate cancer under certain genetic and environmental conditions. Lastly, dysbiosis in the fecal microbiome of ESR1 KO mice might induce systemic effects.

## Methods

### Animals

The animal experiments were approved by the University of Missouri Animal Care and Use Committee (Protocol #7948) and performed in accordance with the recommendations in the Guide for the Care and Use of Laboratory Animals of the National Institutes of Health. Mice were ad libitum fed 5008 Purina Chow (Bourn Feed, Columbia, MO) and provided free access to water. They were maintained on a 12 hr light: 12 hr dark cycle with the lights on at 7.30 hrs and off at 19.30 hrs. To generate WT and ESR1 KO mice, males and females heterozygous for the ESR1 gene were bred. At weaning (3 weeks of age), approximately 1–2 mm section of tail was excised, DNA was isolated, and the genotype status for ESR1 determined as reported previously^64^.

### Collection of fecal and seminal fluid samples

Prior to euthanasia, each animal was placed one per cage without any bedding. Adult males ranging in age from 3 to 13 months of age were used for these studies. Four to five fecal boli were collected from each animal. We collected the fecal samples *ex vivo* to replicate the approach used in similar studies^65^,^66^ and to ensure that the bacterial composition was reflective of the entire gastrointestinal system, including the rectum and anus. The animals were then intra-peritoneally injected with ~0.8 to 0.9 ml of 1.2% concentration of avertin (Sigma-Aldrich, Saint Louis, Missouri). After they were anesthetized, as evidenced by lack of corneal and leg withdrawal reflexes, the fur from the abdominal region extending from the xiphoid process to the pelvic region was shaved with a Wahl Clipper Corp, model 9962 (Wahl, Shelton, CT). The animals were then surgically scrubbed with a combination of 75% BD alcohol swabs (Becton, Dickinson and Company; Franklin Lakes, NJ) and betadine (Equate, Bentonville, Arkansas). The surgically prepped males were placed in a laminar flow-hood (Nuaire NU 407–600, Plymouth, Minnesota) that had been previously cleaned with bleach and 75% EtOH followed by overnight UV radiation. The two experimenters surgically scrubbed their hands with E-Z scrub 205 betadine sponges (Becton, Dickinson and Company; Franklin Lakes, NJ) and darned a sterile surgical gown, gloves, and mask. After the animals were placed in the hood, the abdominal skin and muscles and *linea alba* were incised. The seminal vesicles were excised and placed into a petri dish. The seminal fluid was then gently extruded into a 2.0 ml Corning Cryogenic Vial (Corning Incorporated, Corning, NY). The fecal and seminal fluid samples were placed on dry ice and then stored at −80 °C until the samples were processed.

### Isolation of seminal fluid and fecal microbial DNA

The microbial DNA from seminal fluid samples was isolated with either the Ultra-Deep Microbiome Prep kit (CaerusBio Inc., Downington, PA) or Qiamp DNA microbiome kit (Qiagen, Valencia, CA). Both of these kits destroy and remove any host cells in the process. The fecal microbial DNA was isolated using the PowerFecal DNA Isolation kit (Mo Bio Laboratories, Inc., Carlsbad, CA). Respective protocols for each kit were followed. The quantity of DNA isolated was measured using Qubit 3.0 Fluorometer (Life Technologies, Grand Island, NY). The number of replicates is indicated in Supplementary Table 1.

### 16s rRNA sequencing

The University of Missouri DNA Core Facility prepared bacterial 16S ribosomal DNA amplicons from extracted fecal and seminal fluid DNA by amplification of the V4 hypervariable region of the 16s rDNA with universal primers (U515F/806R) flanked by Illumina standard adapter sequences^67^,^68^. Universal primer sequences are available at proBase (http://www.microbial-ecology.net/probebase/)^69^. A forward primer and reverse primer with a unique 12-base index were used in each PCR reaction. PCR reactions (50 ul) contained 100 ng of genomic DNA, forward and reverse primers (0.2 uM each), dNTPs (200 uM each), and Phusion High-Fidelity DNA Polymerase (1U). PCR amplification was performed as follows: 98 °C(3:00) + [98 °C(0:15) + 50 °C(0:30) + 72 °C(0:30)] × 25 cycles + 72 °C(7:00). Maximum sample volume was added to each PCR reaction for seminal fluid samples which contained <100 ng of input DNA. Amplified product (5 ul) from each PCR reaction was combined and thoroughly mixed to prepare a single pool. Pooled amplicons were then purified by addition of Axygen AxyPrep MagPCR Clean-up beads (50 ul) to an equal volume of 50 ul of the amplicon library pool and incubated at room temperature for 15 minutes. Products were placed on a magnetic stand for 5 minutes and supernatant (95 μl) was removed and discarded. Each well was washed by addition of 200 ul of freshly prepared 80% EtOH, incubation at room temperature for 30 seconds, and removal of supernatant. Wash steps were repeated once and plate was allowed to dry on magnetic stand for 15 minutes. The dried pellet was resuspended in Qiagen EB Buffer (32.5 ul), incubated at room temperature for 2 minutes, and then placed on the magnetic stand for 5 minutes. Supernatant (30 ul) was transferred to low binding microcentrifuge tube for storage. The final amplicon pool was evaluated using the Advanced Analytical Fragment Analyzer automated electrophoresis system, quantified with the Qubit flourometer using the quant-iT HS dsDNA reagent kit (Invitrogen), and diluted according to Illumina’s standard protocol for sequencing on the MiSeq.

### Bioinformatics and amplicon analysis

Paired-end Illumina MiSeq DNA reads were joined using FLASH^70^. Usearch7^71^ was used to clean contigs and remove those with E >0.5, as explained here: http://drive5.com/usearch/manual/exp_errs.html. Contigs were clustered to 97% identity against DNA sequences in the Greengenes database^72^, version 13_5, using the QIIME^73^, version 1.8, script pick_closed_reference_otus.py, which obviates chimera and PCR error detection. For alpha-diversity, Chao1 (species richness) and Shannon (species diversity) values were calculated and plotted using the phlyoSeq R package^74^. Rarefaction metrics were calculated using the alpha_rarefaction.py script in the Qiime package^73^ and plotted using Microsoft Excel (Supplementary Fig. 1).

Measurements of beta-diversity were facilitated by the QIIME script beta_diversity_through_plots.py and visualized using Principal Coordinate Analysis, PCoA, as implemented in QIIME^73^. LEfSe^75^ was used to identify genera most consistently different between sample types. LEfSe results were visualized using taxonomy bar-chart and cladogram plots, as implemented on the LEfSe website, http://huttenhower.sph.harvard.edu/galaxy/. PCoA was used to determine whether age of WT and ESR1 KO mice affected the seminal fluid and fecal microbiome samples, where two age-ranges where compared: <220 and >220 days of age. For the fecal microbiome, metagenomeSeq^47^, version 1.10.0 was used to identify the OTUs in WT and ESR1 KO that varied based on these two age groups. To identify genera associated with the WT and ESR1 KO genotypes within fecal and seminal sample types, we used metagenomeSeq^47^, version 1.10.0. Bacterial metabolic characterization of sample types was facilitated with PICRUSt^76^, version 1.0.0. To correlate the genera changes with metabolic characteristics of sample types, we used a custom R script provided as a gift from Dr. Jun Ma and Kjersti Aagaard-Tillery, Baylor College of Medicine, Houston, TX.

## Additional Information

**How to cite this article**: Javurek, A. B. *et al.* Discovery of a Novel Seminal Fluid Microbiome and Influence of Estrogen Receptor Alpha Genetic Status. *Sci. Rep.* **6**, 23027; doi: 10.1038/srep23027 (2016).

## Supplementary Material

Supplementary Information

Dataset 1

Dataset 2

Dataset 3

## Figures and Tables

**Table 1:** OTUs that differ in the seminal fluid between WT and ESR1 KO mice, as determined by metagenomeSeq.

Bacterial Taxonomy	Log 2 Fold Change (WT vs. ESR1 KO)	Adjusted P Value
Proteobacteria; Gammaproteobacteria; Pseudomonadales; Pseudomonadaceae	−2.30	0.0141
Firmicutes; Bacilli; Lactobacillales; Aerococcaceae; Alloiococcus	−1.90	0.0183
Proteobacteria; Betaproteobacteria; Rhodocyclales; Rhodocyclaceae	−1.63	0.0171
Actinobacteria; Actinobacteria; Actinomycetales; Nocardioidaceae	−1.62	0.0103
Proteobacteria; Alphaproteobacteria; Sphingomonadales; Sphingomonadaceae; Sphingomonas	−1.07	0.0357
Proteobacteria; Gammaproteobacteria; Pseudomonadales; Pseudomonadaceae; Pseudomonas; viridiflava	−0.913	0.0212
Firmicutes; Bacilli; Lactobacillales; Streptococcaceae; Streptococcus	−0.750	0.0062
Bacteroidetes; Sphingobacteriia; Sphingobacteriales; Sphingobacteriaceae; Pedobacter	−0.698	0.0141
Actinobacteria; Actinobacteria; Actinomycetales; Brevibacteriaceae; Brevibacterium	−0.491	0.0162
Actinobacteria; Actinobacteria; Actinomycetales; Actinomycetaceae; Actinomyces	−0.465	0.0167
Proteobacteria; Gammaproteobacteria; Pseudomonadales; Moraxellaceae; Acinetobacter	−0.407	0.0212
Firmicutes; Bacilli; Gemellales; Gemellaceae	−0.383	0.0103
Proteobacteria; Gammaproteobacteria; Xanthomonadales; Xanthomonadaceae	−0.359	0.0483
Firmicutes; Clostridia; Clostridiales; [Tissierellaceae]; Peptoniphilus	−0.328	0.0112
Actinobacteria; Actinobacteria; Actinomycetales; Corynebacteriaceae; Corynebacterium	−0.174	0.0106
Cyanobacteria; Chloroplast; Streptophyta	−0.126	0.0103
Proteobacteria; Betaproteobacteria; Burkholderiales; Alcaligenaceae; Achromobacter	−0.111	0.0099
Firmicutes; Bacilli; Lactobacillales; Streptococcaceae; Lactococcus	0.171	0.0095
Proteobacteria; Gammaproteobacteria; Pseudomonadales; Pseudomonadaceae; Pseudomonas	0.185	0.0095
Proteobacteria; Gammaproteobacteria; Pasteurellales; Pasteurellaceae; Haemophilus; parainfluenzae	0.229	0.0063
Proteobacteria; Gammaproteobacteria; Oceanospirillales; Alcanivoracaceae; Alcanivorax; dieselolei	0.243	0.0095
Firmicutes; Clostridia; Clostridiales; [Tissierellaceae]; Anaerococcus	0.243	0.0062
Proteobacteria; Alphaproteobacteria; Rhizobiales; Methylobacteriaceae; Methylobacterium	0.312	0.0162
Actinobacteria; Actinobacteria; Actinomycetales; Propionibacteriaceae; Propionibacterium; acnes*	0.337	0.0007
Firmicutes; Bacilli; Bacillales; Staphylococcaceae; Staphylococcus; equorum	0.409	0.0162
Firmicutes; Bacilli; Lactobacillales	0.503	0.0063
Firmicutes; Bacilli; Bacillales; Staphylococcaceae; Staphylococcus	0.641	0.0099
Proteobacteria; Gammaproteobacteria; Pseudomonadales; Pseudomonadaceae; Pseudomonas; veronii	0.680	0.0162
Firmicutes; Bacilli; Lactobacillales; Lactobacillaceae; Lactobacillus; iners	0.717	0.0063
Proteobacteria; Betaproteobacteria; Neisseriales; Neisseriaceae;	0.838	0.0316
Proteobacteria; Betaproteobacteria; Burkholderiales; Comamonadaceae;	0.971	0.0103
Firmicutes; Clostridia; Clostridiales; [Tissierellaceae]; Finegoldia;	1.05	0.0171
Proteobacteria; Betaproteobacteria; Burkholderiales; Comamonadaceae; Schlegelella;	1.08	0.0104

Bolded genera are greater in ESR1 KO males; whereas non-bolded genera are more abundant in WT males. *Bacterium associated with chronic prostatitis culminating in prostate cancer in men and rodent models.

**Table 2:** OTUs that differ in the fecal microbiome between WT and ESR1 KO mice, as determined by metagenomeSeq.

Bacterial Taxonomy	Log 2 Fold Change (WT *vs.* ESR1 KO)	Adjusted P Value
Bacteroidetes; Bacteroidia; Bacteroidales; Bacteroidaceae; Bacteroides	−0.488	0.0198
Cyanobacteria; 4C0d-2; YS2	−0.432	0.0054
Bacteroidetes; Bacteroidia; Bacteroidales; Porphyromonadaceae; Parabacteroides	−0.191	0.0025
Firmicutes; Clostridia; Clostridiales; Lachnospiraceae; Coprococcus	−0.147	0.0003
Bacteroidetes; Bacteroidia; Bacteroidales; Rikenellaceae	−0.070	0.0038
Firmicutes; Clostridia; Clostridiales; Lachnospiraceae	−0.049	0.0054
Firmicutes; Clostridia; Clostridiales; Christensenellaceae	−0.030	0.0031
Bacteroidetes; Bacteroidia; Bacteroidales; Bacteroidaceae; Bacteroides; acidifaciens	0.108	0.0249
TM7; TM7-3; CW040; F16	0.140	0.0071
Firmicutes; Clostridia; Clostridiales; Ruminococcaceae	0.145	0.0156
Firmicutes; Clostridia; Clostridiales	0.158	0.0005
Firmicutes; Clostridia; Clostridiales; Dehalobacteriaceae; Dehalobacterium	0.164	0.0087
Firmicutes; Clostridia; Clostridiales; Lachnospiraceae; Dorea	0.240	0.0002
Bacteroidetes; Bacteroidia; Bacteroidales	0.295	0.0038
Proteobacteria; Betaproteobacteria; Burkholderiales; Alcaligenaceae; Sutterella	0.342	0.0071
Firmicutes; Clostridia; Clostridiales; [Mogibacteriaceae]	0.372	0.0054
Firmicutes; Clostridia; Clostridiales; Ruminococcaceae; Oscillospira	0.478	0.0075
Proteobacteria; Alphaproteobacteria; Rickettsiales; mitochondria	0.551	0.0156
Bacteroidetes; Bacteroidia; Bacteroidales; S24-7	0.617	0.0038
Firmicutes; Clostridia; Clostridiales; Clostridiaceae	0.747	0.0249
Firmicutes; Bacilli; Turicibacterales; Turicibacteraceae; Turicibacter	1.09	0.0038
Firmicutes; Erysipelotrichi; Erysipelotrichales; Erysipelotrichaceae; Allobaculum	1.52	0.0038
Actinobacteria; Actinobacteria; Bifidobacteriales; Bifidobacteriaceae; Bifidobacterium; pseudolongum	2.46	0.0038

Bolded genera are greater in ESR1 KO males; whereas non-bolded genera are more abundant in WT males.
