# Corrigendum: Discovery of a Novel Seminal Fluid Microbiome and Influence of Estrogen Receptor Alpha Genetic Status

**DOI:** 10.1038/srep25216

**Published:** 2016-04-29

**Authors:** Angela B. Javurek, William G. Spollen, Amber M. Mann Ali, Sarah A. Johnson, Dennis B. Lubahn, Nathan J. Bivens, Karen H. Bromert, Mark R. Ellersieck, Scott A. Givan, Cheryl S. Rosenfeld

Scientific Reports 6: Article number: 23027; 10.1038/srep23027 published online: 03142016; updated: 04292016.

In this Article, Figure 4 is a duplication of Figure 6. The correct Figure 4 appears below as Figure 1.

## Figures and Tables

**Figure 1:**
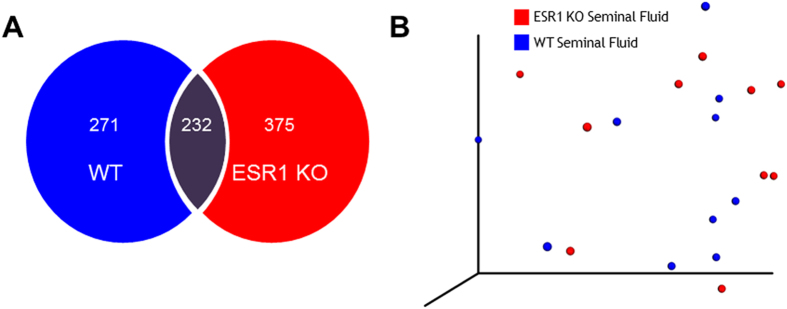
Figure 1.

