# Supplementary material for: Discovery of a Novel Seminal Fluid Microbiome and Influence of Estrogen Receptor Alpha Genetic Status
Source: Sci Rep. 2016 Mar 14;6:23027. doi: 10.1038/srep23027 (PMC4789797; doi:10.1038/srep23027)
Supplement: Supplementary Information [file srep23027-s1.docx]

**Supplementary Information**

**Discovery of a Novel Seminal Fluid Microbiome and Influence of**

**Estrogen Receptor Alpha Genetic Status**

**Angela B. Javurek^1,2^, William G. Spollen^1,3^, Amber M. Mann Ali^4,5^, Sarah A. Johnson^1,2,6^, Dennis B. Lubahn^4,5,7,8^, Nathan J. Bivens^9^, Karen H. Bromert^9^, Mark R. Ellersieck^10^,**

**Scott A. Givan^1,3,11^, and Cheryl S. Rosenfeld^1,2,8,12^**

**Departments of:**

^1^Bond Life Sciences Center, University of Missouri, Columbia, MO 65211 USA

^2^Biomedical Sciences, University of Missouri, Columbia, MO 65211 USA

^3^Informatics Research Core Facility, University of Missouri, Columbia, MO 65211 USA

^4^Biochemistry, University of Missouri, Columbia, MO 65211 USA

^5^MU Center for Botanical Interaction Studies, University of Missouri, Columbia, MO 65211 USA

^6^Animal Sciences, University of Missouri, Columbia, MO 65211 USA

^7^Child Health, University of Missouri, Columbia, MO 65211 USA

^8^Genetics Area Program, University of Missouri, Columbia, MO 65211 USA

^9^DNA Core Facility, University of Missouri, Columbia, MO 65211 USA

^10^Agriculture Experimental Station-Statistics, University of Missouri, Columbia, MO 65211 USA

^11^Molecular Microbiology and Immunology, University of Missouri, Columbia, MO 65211 USA

^12^Thompson Center for Autism and Neurobehavioral Disorders, University of Missouri, Columbia, MO 65211 USA

**Short Title: The Seminal Fluid Microbiome**

**Keywords:** Male, reproductive tract, semen, bacteria, metabolic pathway, gut microbiome, DOHaD, and transgenic mice

**Correspondence:** Cheryl S. Rosenfeld, DVM, PhD, Biomedical Sciences and Bond Life Sciences Center, University of Missouri, 440F Bond Life Sciences Center, 1201 E. Rollins Rd., Columbia, MO 65211. E-email: [rosenfeldc@missouri.edu](mailto:rosenfeldc@missouri.edu), phone: (573) 882-5132, and fax: (573) 884-9395

**Supplementary Table**

| **Supplementary Table 1**. OTUs that differ in the fecal samples between young and older adults, as determined by metagenomeSeq. Shaded ones are greater in older animals; whereas non-shaded ones are more abundant in younger animals. | | |
| --- | --- | --- |
| **Bacterial Taxonomy** | **Log 2 Fold Change (WT vs ESR 1 KO)** | **Adjusted**  **P value** |
| Firmicutes; Bacilli; Lactobacillales; Lactobacillaceae; Lactobacillus | -3.022 | 0.0009 |
| Bacteria; Firmicutes; Clostridia; Clostridiales; Peptostreptococcaceae | -2.804 | 0.0009 |
| Actinobacteria; Actinobacteria; Bifidobacteriales; Bifidobacteriaceae; Bifidobacterium; pseudolongum | -2.45 | 0.002 |
| Firmicutes; Clostridia; Clostridiales; Clostridiaceae | -2.045 | 0.005 |
| Tenericutes; Mollicutes; RF39 | -1.741 | 0.05 |
| Firmicutes; Clostridia; Clostridiales; Clostridiaceae; Clostridium; | -1.644 | 0.008 |
| Firmicutes; Bacilli; Turicibacterales; Turicibacteraceae; Turicibacter | -1.253 | 0.002 |
| TM7; TM7-3; CW040 | -1.137 | 0.0009 |
| Firmicutes; Clostridia; Clostridiales; Lachnospiraceae; Dorea | -1.027 | 8.73 X10^-6^ |
| Firmicutes; Clostridia; Clostridiales; Clostridiaceae; Sarcina | -1.01 | 0.04 |
| Firmicutes; Clostridia; Clostridiales; Mogibacteriaceae | -1.004 | 0.0009 |
| Cyanobacteria; 4C0d-2; YS2 | -0.957 | 0.002 |
| Bacteroidetes; Bacteroidia; Bacteroidales; | -0.676 | 0.002 |
| Proteobacteria; Betaproteobacteria; Burkholderiales; Alcaligenaceae; Sutterella; | -0.491 | 0.009 |
| Firmicutes; Erysipelotrichi; Erysipelotrichales; Erysipelotrichaceae; Allobaculum | -0.407 | 0.007 |
| Firmicutes; Clostridia; Clostridiales; Christensenellaceae | -0.313 | 0.0009 |
| Bacteroidetes; Bacteroidia; Bacteroidales; Rikenellaceae | -0.0426 | 0.002 |
| Proteobacteria; Alphaproteobacteria; Rickettsiales; mitochondria | -0.023 | 0.01 |
| Firmicutes; Clostridia; Clostridiales | 0.012 | 0.0004 |
| Firmicutes; Clostridia; Clostridiales; Ruminococcaceae | 0.0898 | 0.009 |
| Firmicutes; Clostridia; Clostridiales; Ruminococcaceae; Ruminococcus | 0.229 | 0.04 |
| Firmicutes; Clostridia; Clostridiales; Lachnospiraceae; Coprococcus | 0.247 | 0.0002 |
| Bacteroidetes; Bacteroidia; Bacteroidales; Porphyromonadaceae; Parabacteroides | 0.407 | 0.0009 |
| Firmicutes; Clostridia; Clostridiales; Lachnospiraceae | 0.537 | 0.002 |
| Firmicutes; Clostridia; Clostridiales; Dehalobacteriaceae; Dehalobacterium | 0.616 | 0.002 |
| Bacteroidetes; Bacteroidia; Bacteroidales; Bacteroidaceae; Bacteroides; acidifaciens | 0.848 | 0.009 |
| Bacteroidetes; Bacteroidia; Bacteroidales; Bacteroidaceae; Bacteroides | 1.214 | 0.007 |
| Bacteroidetes; Bacteroidia; Bacteroidales; S24-7 | 1.258 | 0.0009 |
| Firmicutes; Clostridia; Clostridiales; Ruminococcaceae; Oscillospira | 2.1 | 0.0004 |
| Firmicutes; Clostridia; Clostridiales; Peptococcaceae | 2.809 | 0.0009 |

**Supplementary Figures**

**
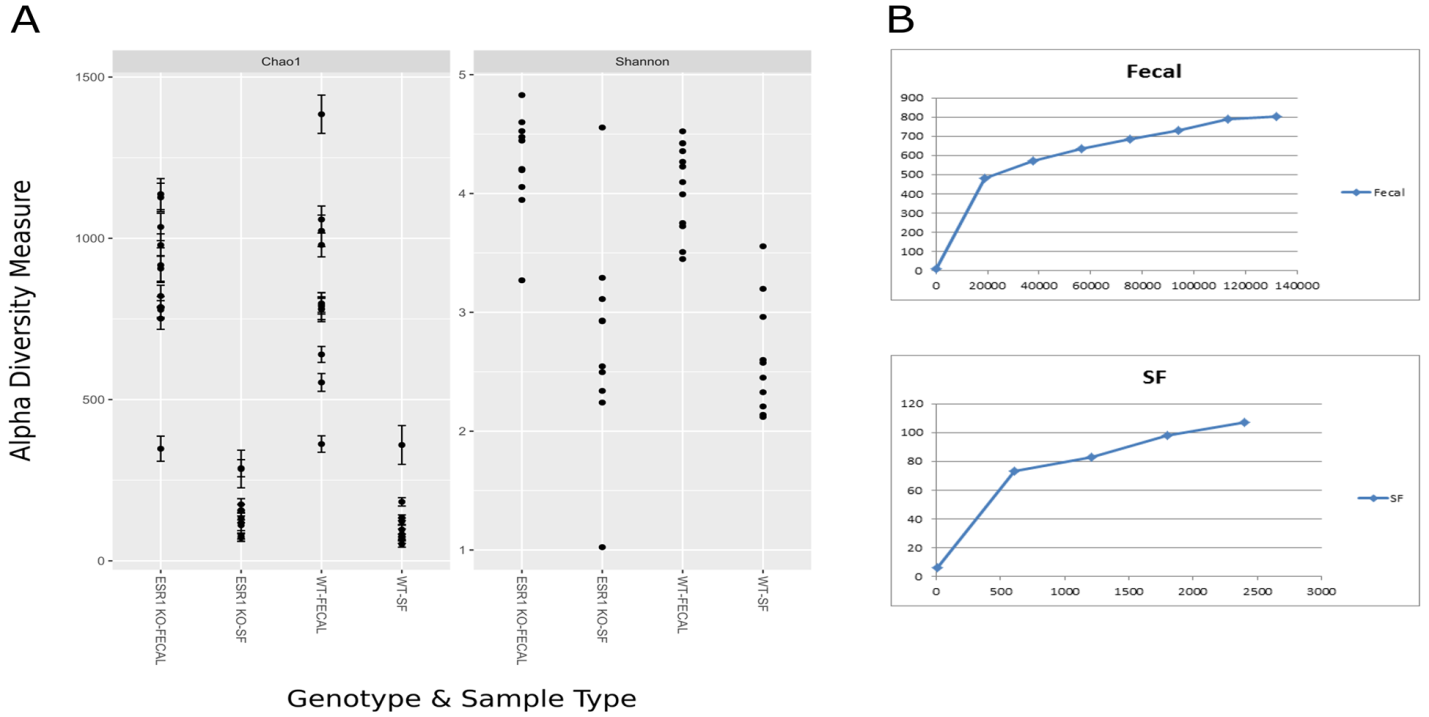
**

**Supplementary Figure 1.** Alpha-diversity analyses of fecal and seminal fluid (SF) samples. A) Species richness (Chao1) and diversity (Shannon) indices of ESR1 KO Fecal, ESR1 KO SF, WT Fecal, and WT SF plotted using the phyloSeq R package plot_richness function ^74^. B) Rarefaction metrics plotted for aggregate fecal and SF samples using the alpha_rarefaction.py script in the Qiime package ^73^.

**
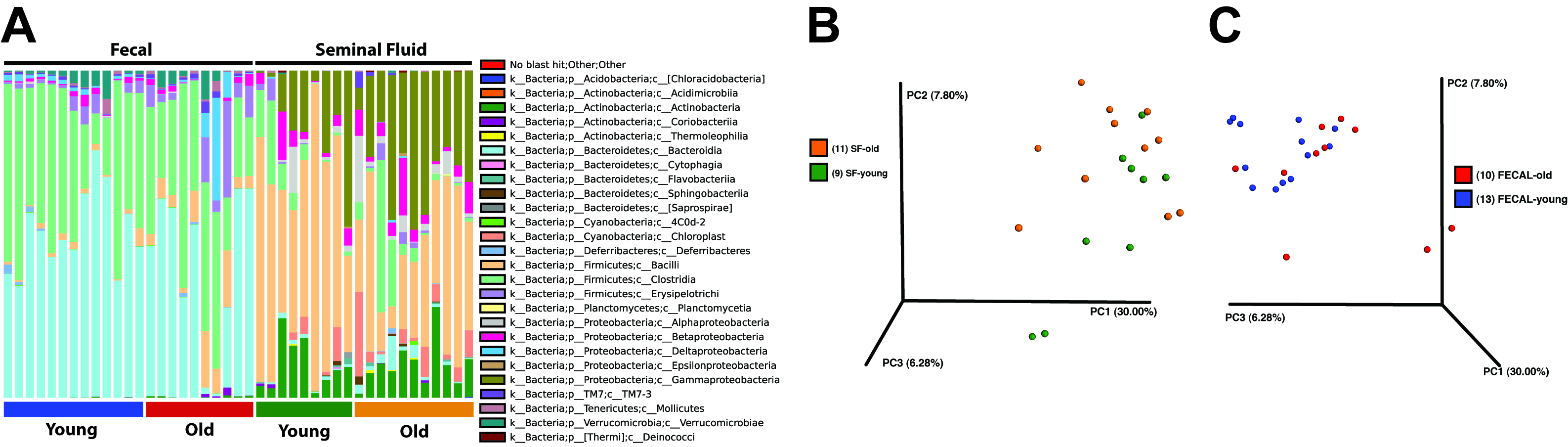
**

**Supplementary Figure 2.** Effect of age on the seminal fluid and fecal microbiomes. A) Bar plot of the most abundant bacterial classes between fecal and SF samples in both WT and ESR1 KO mice organized based on age of the animals. B) PCoA of the SFM based on age (< 220 or > 220 days of age; p = 0.4 by PERMANOVA). C) PCoA of the fecal microbiome based on age (< 220 or > 220 days of age; p = 0.01 by PERMANOVA).
